# Illumina MiSeq sequencing disfavours a sequence motif in the GFP reporter gene

**DOI:** 10.1038/srep26314

**Published:** 2016-05-19

**Authors:** Silvie Van den Hoecke, Judith Verhelst, Xavier Saelens

**Affiliations:** 1 Medical Biotechnology Center, VIB, Ghent, B-9052, Belgium; 2 Department of Biomedical Molecular Biology, Ghent University, Ghent, B-9052, Belgium

## Abstract

Green fluorescent protein (GFP) is one of the most used reporter genes. We have used next-generation sequencing (NGS) to analyse the genetic diversity of a recombinant influenza A virus that expresses GFP and found a remarkable coverage dip in the GFP coding sequence. This coverage dip was present when virus-derived RT-PCR product or the parental plasmid DNA was used as starting material for NGS and regardless of whether Nextera XT transposase or Covaris shearing was used for DNA fragmentation. Therefore, the sequence coverage dip in the GFP coding sequence was not the result of emerging GFP mutant viruses or a bias introduced by Nextera XT fragmentation. Instead, we found that the Illumina MiSeq sequencing method disfavours the ‘CCCGCC’ motif in the GFP coding sequence.

Influenza viruses are important human and animal pathogens that have evolved numerous mechanisms to subvert their host’s innate and adaptive immune system. Recombinant influenza viruses that express a reporter gene are thus very useful to study viral replication, spread and cell tropism *in vitro* and *in vivo.* The use of such reporter viruses can also facilitate the discovery of new antivirals and vaccines^1^,^2^,^3^,^4^. However, adding a reporter gene to the relatively small influenza virus genome has no selective advantage for the virus. Instead, influenza viruses expressing a reporter gene are attenuated compared to their parental counterparts^5^,^6^,^7^,^8^,^9^. Influenza viruses that have lost (part of) the reporter gene can thus quickly outgrow the original reporter virus. Such reporter gene loss can *e.g*. lead to false negative hits in a compound screening experiment that is based on reporter gene expression as a read out. It is therefore important to study the genomic stability of the viral population derived from a recombinant influenza virus clone. Next-generation sequencing (NGS) is very well suited to determine the genomic stability of recombinant influenza viruses due to its high sequencing output (up to hundreds of Gigabases)^10^,^11^. In addition, the small genomic size, approximately 14,000 bases of negative stranded RNA, of influenza viruses enables sequencing of viral samples at high coverage for each position in the genome. We recently optimized an influenza RT-PCR protocol and NGS data analysis pipeline to study the genomic composition of an influenza A virus population^12^.

We previously reported the generation and characterization of a recombinant influenza A virus that expresses GFP^1^. In that virus, named PR8-NS1(1-73)GFP, the GFP transgene is encoded by the middle part of a tri-cistronic gene segment 8^1^. The virus is phenotypically stable and appeared to be genetically stable based on lllumina MiSeq sequencing of full genome RT-PCR products of this virus^1^. However, we noticed a twofold drop in sequence coverage within the GFP coding sequence (Fig. 1, orange line)^1^. This coverage dip could be the result of different processes. First, a proportion of the PR8-NS1(1-73)GFP progeny virus might have lost part of the GFP coding sequence. Second, sequence preference of the transposase-based Nextera XT fragmentation could account for the sequence coverage dip^13^,^14^,^15^. A less favourable sequence motif in the GFP coding region could lead to a fragmentation bias and hence lower sequence coverage^13^,^14^,^15^,^16^. The ‘Illumina Nextera XT DNA library preparation kit’ fragments the DNA and adds the desired sequencing adaptors in a single step by using a transposition reaction, a process that is named tagmentation^13^. The transposase is target sequence based and, as a consequence, near-random^13^,^14^,^15^,^16^,^17^. Finally, sequencing bias by the Illumina MiSeq sequencer itself, due to a motif in the GFP sequence, could explain the drop in sequence coverage that we observed. It has been shown that the Illumina MiSeq exhibits sequencing biases for different sequence types, *e.g*. in regions with a low or high GC-content, long homopolymers or inverted repeats^17^,^18^,^19^.

Here, we report that a sequence motif in the GFP coding sequence leads to a significant reduction in sequence coverage when using Illumina MiSeq sequencing. This finding is important for NGS analysis of small microbial genomes, in particular when GFP is included as a reporter in those genomes.

## Results

### An NGS coverage dip in the GFP sequence irrespective of the fragmentation method used

**Figure 1:**
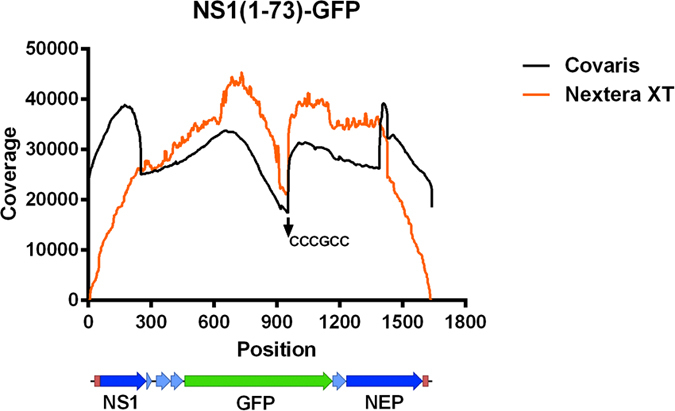
Sequence coverage of the viral NS1(1-73)-GFP segment. Sequence coverage as determined by Illumina MiSeq sequencing after Nextera XT (orange) or Covaris (black) fragmentation and CLC Genomics Workbench version 7.0.3 data processing. The obtained sequences were filtered, trimmed and mapped to the reference sequence of the NS1(1-73)-GFP segment (based on the plasmid used to generate the recombinant PR8-NS1(1-73)GFP virus, with addition of the extra 20 nucleotides present at the 5′ site in the RT-PCR primers)^12^. Below sequencing coverage plot: schematic representation of NS1(1-73)-GFP segment.

We previously used Nextera XT tagmentation followed by NGS on an Illumina MiSeq sequencing platform to study the genetic heterogeneity of a GFP-expressing influenza A virus^1^. Sequence coverage was high and homogenous for all eight virus genome segments, with the exception of the 5′ and 3′ termini^1^. The latter is expected when using a transposon-based fragmentation technique to make a library of fragments derived from a discrete set of relatively small linear double stranded DNA molecules^12^,^13^. However, we noticed a twofold drop in sequence coverage from over 40,000 to almost 20,000 reads per position near the middle of the NS1(1-73)-GFP segment (Fig. 1, orange line)^1^. The PR8-NS1(1-73)GFP virus retained GFP expression over multiple rounds of replication *in vitro*, suggesting that the coverage dip was unlikely the result of the rapid evolution of a subpopulation of viruses that had lost part of the GFP information^1^. We first explored the possibility that this apparent coverage dip could be the result of the sequence dependency of Nextera XT fragmention, which has a known target sequence bias^13^,^14^,^15^. Therefore, we repeated the Illumina MiSeq NGS analysis of the PR8-NS1(1-73)GFP virus using Covaris shearing for the library preparation. This is a mechanical shearing technique that is based on adaptive focused acoustics, and therefore more random. We used the same RT-PCR sample of the PR8-NS1(1-73)GFP virus which had been sequenced previously on the Illumina MiSeq after Nextera XT fragmentation^1^. Mapping of the reads to the PR8-NS1(1-73)GFP reference genome resulted in high coverage across all eight segments, which now also included the genome segment ends (Supplementary Figure S1). However, we again observed a decrease in coverage at the same position in the GFP coding sequence (nucleotide position: 452–1162, with the lowest coverage at position 952; Fig. 1, black line), similar to the one observed after Nextera XT fragmentation. This indicates that this dip is not caused by the sequence dependency of the transposition reaction in the Nextera XT fragmentation. We note that the ends of the viral fragments are overrepresented after Covaris fragmentation because adaptors are ligated to mechanically sheared DNA, a process that is favored at the free ends of the influenza genome^12^.

The above results do not exclude the possibility that viruses with a deletion in the GFP coding sequence were present in the virus population that we used as starting material. We investigated the presence of major deletions in the GFP sequence by using the ‘CLC Genomics Workbench Large Gap Mapper’, which aligns reads to the reference sequence, while allowing large gaps in the mapping. Based on the ‘Large Gap Mapper’ 0.22% more reads were aligned to the reference genome, compared to regular mapping. The distribution of these extra mapped reads over the eight segments ranged from 0.04% (M segment) to 0.60% (PA segment). An increase of 0.41% of mapped reads was recorded for the NS1(1-73)-GFP segment. Therefore, fragments with a large deletion were not substantially enriched for the NS1(1-73)-GFP segment, indicating that the dip in coverage was not caused by large deletions in the GFP sequence.

### NGS sequencing of the pHW-NS1(1-73)Dmd-GFP-NEP plasmid also reveals a coverage dip

**Figure 2:**
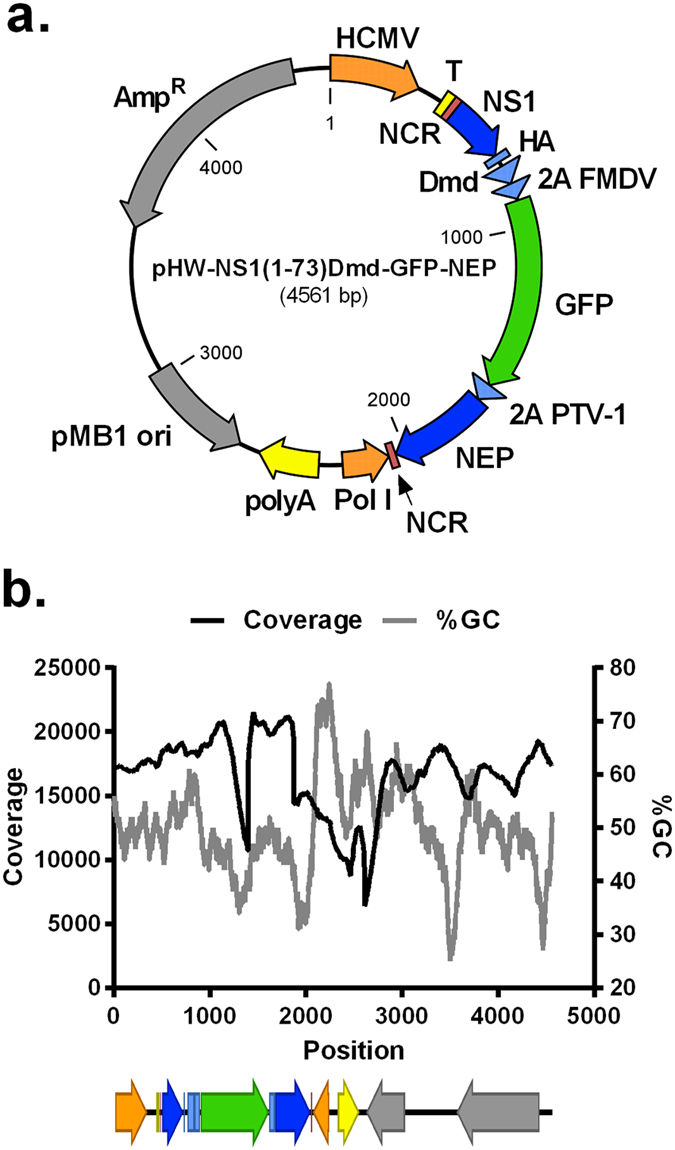
Sequence coverage of pHW-NS1(1-73)Dmd-GFP-NEP based on Illumina MiSeq sequencing. (**a**) Map of pHW-NS1(1-73)Dmd-GFP-NEP. (**b**) Sequence coverage (black line) and GC-percentage distribution (grey line, window size: 100) of the pHW-NS1(1-73)Dmd-GFP-NEP plasmid as determined by Illumina MiSeq sequencing after Covaris shearing and CLC Genomics Workbench version 7.0.3 data processing^12^. The diagram below the graph shows the organization of the different features from position 1 to position 4561 in the linearized pHW-NS1(1-73)Dmd-GFP-NEP plasmid. HCMV: human cytomegalovirus promoter, T: terminator sequence, NCR: non-coding region, NS1: non-structural protein 1, HA: hemagglutinin-tag, Dmd: dimerization domain (Dmd) of the *Drosophila melanogaster* Ncd protein, 2A FMDV: foot-and-mouth disease virus-2A auto processing site, 2A PTV-1: porcine teschovirus-1 2A cleavage site, NEP: nuclear export protein, Pol I: human RNA polymerase I promoter, polyA: polyA terminator, pMB1 ori: origin of replication, Amp^R^: ampicillin resistance gene.

Based on the above analyses it is unlikely that the observed variability in the PR8-NS1(1-73)GFP virus population was responsible for the coverage dip in GFP. We therefore hypothesized that the Illumina MiSeq platform caused the coverage bias in the GFP coding sequence. To test this, we sequenced the pHW-NS1(1-73)Dmd-GFP-NEP plasmid that was used to generate the GFP expressing influenza A virus. In this way, we could also assess a possible effect of RT-PCR efficacy on the sequencing coverage of the GFP sequence. A mean sequencing coverage of 16,655 (+/−2,927) was obtained, with the coverage per position ranging from 6,376 (position 2,612) to 21,513 (position 1,451). We observed a twofold drop in nucleotide coverage in the GFP coding sequence of the plasmid, with the lowest coverage being 10,683 at position 1,395 (Fig. 2).

It has been reported that the performance of Illumina MiSeq sequencing is reduced in regions that have a high or low GC-content^18^,^20^. However, the GFP coding sequence is slightly GC-poor (average 43.18%), compared to the overall GC-percentage of the plasmid (49.55%) (Fig. 2). Based on the relation between the GC-content and sequencing coverage at each position, we conclude that there was no strict correlation between GC-content and sequencing coverage.

Before sequencing on the Illumina MiSeq platform, the DNA fragments are ligated on the sequencing chip through their adaptors and subjected to bridge amplification PCR. Presuming that bridge amplification PCR occurs less efficiently when secondary structures are present in the template, the minimal energy to form secondary structures of fragments of 350 bp (approximately the mean DNA fragment length), with a sliding window of 50 bp was calculated using mFold^21^. This minimal energy is inversely correlated with the formation of secondary structures: the lower the minimal energy needed to form a secondary structure, the higher the chance that this structure will be formed. The mFold calculation predicted that the minimal energy required to form secondary structures is not lower in the GFP coding sequence than the average minimal energy to form secondary structures in the pHW-NS1(1-73)Dmd-GFP-NEP sequence (data not shown). This suggests that the GFP sequence is not more prone to form secondary structures compared to the rest of the plasmid sequence. The dip in coverage in the GFP sequence can therefore not be explained by a less efficient bridge amplification of the fragments containing the GFP sequence.

**Figure 3:**
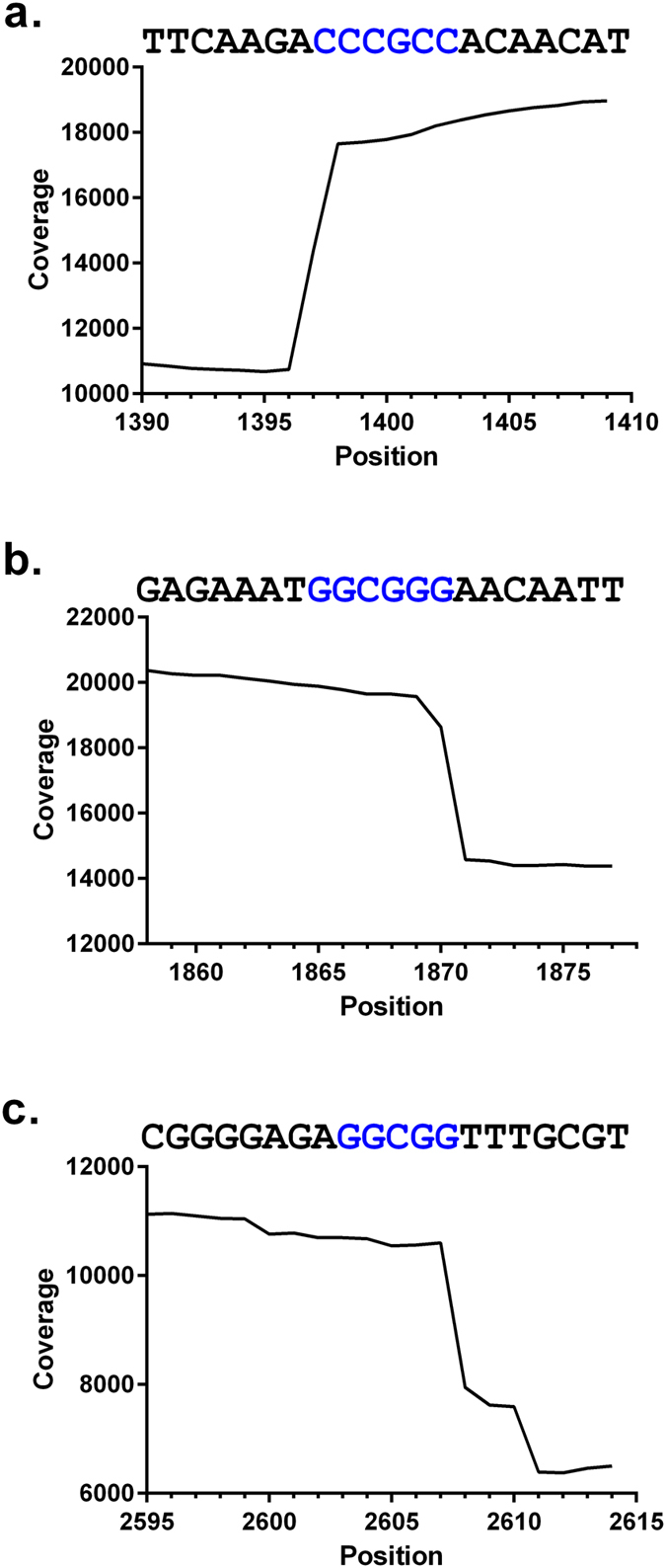
Drop in sequencing coverage at the ‘GGCNGG’ or ‘GGCNG’ motifs. The sequencing coverage is plotted in function of the nucleotide position in the pHW-NS1(1-73)Dmd-GFP-NEP plasmid. The presence of the 'CCCGCC’ motif (reverse complement of ‘GGCGGG’) at position 1397–1402 (**a**), the ‘GGCGGG’ motif at position 1865–1870 (**b**) and the ‘GGCGG’ motif at position 2603–2607 (**c**), result in a drop in sequencing coverage.

Sequence-specific errors were previously reported to be common in Illumina HiSeq reads, with the highest error rates seen at the ‘GGC’ motif, and in particular at ‘GGCNG’^22^. In addition, Ekblom *et al*. observed a negative correlation between the site specific sequencing error rate and the sequencing coverage^19^. In particular, they observed a steep drop in coverage exactly at and upstream of the error prone motif ‘CCNGCC’ (or downstream of its reverse complement ‘GGCNGG’)^19^. This ‘CCNGCC’ motif occurs 12 times in pHW-NS1(1-73)Dmd-GFP-NEP. At two of these motifs (positions 1,397–1,402 and 1,865–1,870) a drop in sequencing coverage is observed. Position 1,397–1,402 is within the GFP coding sequence. At position 1,396 (one nucleotide upstream of the 1,397–1,402 ‘CCCGCC’ motif), there was a drop in sequencing coverage from 14,384 (position 1,397) to 10,744 (position 1,396; Fig. 3a). The ‘GGCGGG’ motif at position 1,865–1,870 was associated with a similar steep sequencing coverage dip (coverage of 18,639 at position 1,870 to a coverage of 14,576 at position 1,871; Fig. 3b). Finally, the error-prone ‘GGCGG’ motif at position 2,603–2,607 was also associated with a drop in coverage from 10,604 at position 2,607 to 7,947 at position 2,608 (Fig. 3c). We manually inspected the quality trimmed reads with unaligned ends at the ‘CCCGCC’ motif and found that part of these reads contained unaligned (mainly single) nucleotides at this position. We also inspected the reads manually prior to quality control trimming, *i.e.* with only the adaptor removed. This analysis revealed that most of the reverse reads were of a too low quality at the nucleotides next to the ‘CCCGCC’ motif and were thus removed during trimming on base quality. Therefore, the steep coverage drop next to the ‘CCCGCC’ motif results from a combination of poor quality at the error prone motif and actual loss of coverage immediately after the motif.

Since multiple different GFP variants are used to generate reporter RNA viruses, we sequenced four plasmids that encode other GFP variants (Supplementary Figure S2)^23^,^24^. A drop in coverage at ‘CCNGCC’ or the shorter ‘CNGCC’ motif can also be observed in some of the sequences encoding these GFP variants^23^,^24^. Although the ‘CCNGCC’ motif is absent in the *Aequorea victoria* GFP coding sequence, the shorter ‘CNGCC’ motif occurs two times in this sequence (positions 820 to 824 and 975 to 979, plasmid numbering). Mapping the reads to the plasmid reference sequence did not reveal a steep drop in sequencing coverage. However, the GFP sequence is not homogenously covered, with the sequencing coverage ranging from 75,529 (minimum; position 1,048) to 108,654 (maximum; position 722). Nevertheless, we found two sharp drops in coverage outside the *A. victoria* GFP coding sequence: upstream of a ‘CCNGCC’ motif at positions 425 to 430 and downstream of a ‘GGCNG’ motif at positions 1,704 to 1,708. From the sequenced GFP variants, the largest loss in coverage in the GFP coding sequence is present in eGFP: a ‘CCGCC’ motif (positions 2,992–2,996) results in a coverage drop of 42,730 at position 2,992 to 32,146 at position 2,991. The MaxGFP/TurboGFP that was used in the NS1-GFP influenza virus reported by Manicassamy *et al.,* displays only a minor sequencing drop (Supplementary Figure S2.c)^5^.

**Figure 4:**
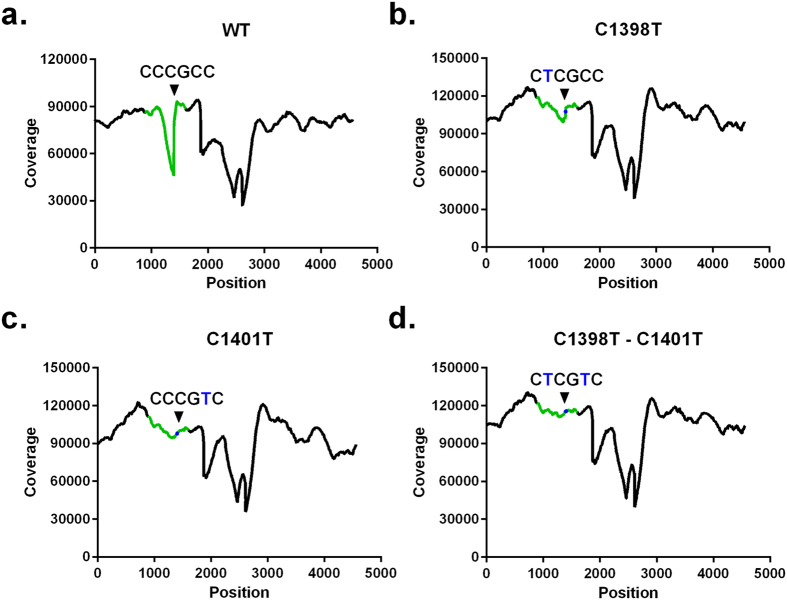
Introducing silent mutations (C1398T and/or C1401T) that interrupt the ‘CCCGCC’ motif in the GFP coding sequence abrogates the drop in sequence coverage. The sequencing coverage is plotted in function of the nucleotide position in pHW-NS1(1-73)Dmd-GFP-NEP (**a**), pHW-NS1(1-73)Dmd-GFP-NEP C1398T (**b**), pHW-NS1(1-73)Dmd-GFP-NEP C1401T (**c**) or pHW-NS1(1-73)Dmd-GFP-NEP C1398T-C1401T (**d**) plasmid. Illumina MiSeq sequencing was performed after Covaris shearing and followed by CLC Genomics Workbench version 7.0.3 data processing and mapping of the reads to the plasmid reference sequence^12^. The position of the CCCGCC (**a**), CTCGCC (**b**), CCCGTC (**c**) and CTCGTC (**d**) motif is marked with an arrow head and the introduced mutation is marked in blue in this motif. The position of the sequence that codes for GFP is marked on the coverage plots in green.

To provide evidence that the observed dip in coverage is a consequence of the presence of the ‘CCCGCC’ motif in the GFP coding region, two silent mutations (separate or in combination) were introduced in pHW-NS1(1-73)Dmd-GFP-NEP: C1398T (resulting in CTCGCC), C1401T (resulting in CCCGTC) and the double mutant C1398T - C1401T (resulting in CTCGTC). The two single mutations in the ‘CCCGCC’ motif largely abolished and the double mutant completely overcame the sequence coverage drop following Illumina MiSeq sequencing (Fig. 4). We can thus conclude that the observed drop in sequencing coverage in the GFP coding region can be linked to the ‘CCCGCC’ motif and that this drop can be eliminated by mutating this motif.

## Discussion

NGS analysis is a powerful tool to study nucleotide sequence variation in biological samples. Ideally, such analysis should result in high and unbiased nucleotide coverage across the target region(s) to provide an accurate picture of the real ratio of sequences present. Uneven coverage of sequences can result in the false interpretation of data, *e.g*. as has been reported for transcriptomics analysis^20^,^25^,^26^.

In general, RNA viruses have a relatively high mutation and recombination rate^27^,^28^. NGS analysis of the genome diversity of certain RNA viruses, such as influenza A, is used to detect escape mutations after antiviral treatment or host immunity and to study the viral population dynamics^29^,^30^,^31^,^32^. In addition, Influenza A viruses with various reporter genes have been generated to facilitate the study of immune responses and cell tropism *in vivo*^1^,^5^,^6^,^33^. Because these studies rely on monitoring the reporter gene products, it is very important to be able to rely on a genetically stable reporter virus. Because the reporter gene does not have a selective advantage for the virus its (partial) deletion in the progeny virus would in most cases offer a competitive advantage over the parental virus. NGS enables sequencing of many viral genomes in a viral population at once. Mapping of these sequencing reads to the reference genome results in a coverage plot, which provides information on the genomic stability of a viral population.

We previously reported on the genomic stability of a GFP expressing influenza A virus that we generated in our lab^1^. Nextera XT tagmentation and Illumina MiSeq sequence analysis of this PR8-NS1(1-73)GFP virus revealed a clear coverage dip in the GFP sequence, which was puzzling because we found that the virus was phenotypically stable over multiple generations^1^. Here, we identified the cause of this GFP-associated coverage drop. This dip was equally apparent when the same sample was analysed after Covaris fragmentation, so it could not be attributed to a sequence preference of the Nextera XT transposase. We also excluded that large deletions in the GFP sequence in the viral population were responsible for the reduced coverage, since NGS analysis of the parental pHW-NS1(1-73)Dmd-GFP-NEP plasmid revealed a similar coverage dip at the same position in the GFP coding sequence. We identified a ‘CCNGCC’ motif in the GFP coding sequence next to the steep drop in coverage. This motif was recently reported to be associated with more errors in the reads generated by Illumina sequencing^19^. The observed coverage dip in the NS1(1-73)-GFP segment is thus the result of a sequencing bias of the Illumina MiSeq for this ‘CCNGCC’ motif.

This work shows that caution is needed when analysing samples containing the GFP sequence by NGS. To avoid this sequencing bias a Quantum SuperGlo GFP coding sequence with silent mutations at positions C504T and/or C507T (GFP numbering) should be used. The ‘CCNGCC’ sequence motif is also present in other GFP versions, *e.g*. the Emerald and ZsGreen1 GFP, which also have been used to generate reporter RNA viruses^34^,^35^,^36^,^37^. The MaxGFP (also named TurboGFP) that was used in the NS1-GFP influenza virus reported by Manicassamy *et al.,* displays only a minor sequencing drop (Supplementary Figure S2.c)^5^.

When designing reporter viruses it is thus important to take into account that there could be a sequencing bias against the reporter gene used. To prevent such an Illumina MiSeq sequencing bias, it is worthwhile to avoid the presence of the error prone ‘CCNGCC’ motif in the reporter gene. In the reported PR8-NS1(1-73)GFP virus, this sequencing bias could lead to the false conclusion that the reporter virus is genetically diverse at the GFP coding sequence.

## Conclusion

We report a striking variation in coverage depth in the GFP sequence of the PR8-NS1(1-73)GFP virus, as analysed by Illumina MiSeq sequencing. We investigated the different sources that could be responsible for this reduced sequencing coverage and found that a ‘CCNGCC’ motif in the GFP coding sequence was the cause of the steep drop in sequencing coverage. Since Illumina MiSeq is the most popular NGS platform that is currently used and GFP is widely used as a reporter gene, we believe that this finding is of value for other researchers, in particular for those instances where genetic variability in concert with GFP reporter gene expression are studied.

## Methods

### Plasmids

The cloning strategy used to construct the pHW-NS1(1-73)Dmd-GFP-NEP plasmid has been described in De Baets *et al*.^1^. The C1398T and/or C1401T mutations were introduced by QuickChange site-directed mutagenesis (Stratagene). The plasmids were transformed and amplified in *Escherichia coli* DH5α. Plasmid DNA was isolated with the Plasmid Midi Kit (Qiagen) according to the manufacturer’s instructions. The sequence of NS1(1-73)Dmd-GFP-NEP and the introduced C1398T or/and C1401T mutations were confirmed by Sanger sequencing on a capillary sequencer (Applied Biosystems 3730XL DNA Analyzer). Plasmids pBluAGFP^24^, pEF6-turboGFP-MCS, pLVX-EF1a-IRES-ZsGreen1 (Clontech-BD Biosciences, Palo Alto, United States) and pDG2-hRIPK4-WT-EGFP-puro^23^ were kindly provided by the BCCM/LMBP Plasmid Collection, Dr. Jens Staal and Giel Tanghe from our Department.

### Cell lines

MDCK, MDCK.PIV5V and HEK293T cells were cultured in DMEM supplemented with 10% FCS, non-essential amino acids, 2 mM L-glutamine, 0.4 mM sodium-pyruvate, 100 U/ml penicillin and 0.1 mg/ml streptomycin at 37 °C in 5% CO2. MDCK cells stably expressing the type I IFN antagonist Paramyxovirus Simian Virus 5 V protein (MDCK.PIV5V) were kindly provided by Dr. Rick Randall (University of St. Andrews, United Kingdom)^38^,^39^. These cell lines were used to rescue and grow PR8-NS1(1-73)GFP virus.

### Production of recombinant viruses

Recombinant influenza virus PR8-NS1(1-73)-GFP was rescued using the A/Puerto Rico/8/34 based reverse genetics system^40^. To generate recombinant PR8-NS1(1-73)-GFP virus, 1 μg of each pHW-plasmid (pHW191-PB2, pHW192-PB1, pHW193-PA, pHW194-HA, pHW195-NP, pHW196-NA, pHW197-M and pHW-NS1(1-73)Dmd-GFP-NEP) was transfected in a HEK293T/MDCK coculture using calcium phosphate precipitation in Optimem. After 36 h, TPCK-treated trypsin (Sigma) was added to a final concentration of 2 μg/ml. After 72 h, the medium was collected. The virus in the medium was amplified on MDCK.PIV5V cells in serum-free cell culture medium in the presence of 2 μg/ml TPCK-treated trypsin (Sigma).

### RT-PCR on PR8-NS1(1-73)GFP virus

Total RNA was isolated from 2 × 10^5^ PFU of PR8-NS1(1-73)GFP virus with the High Pure RNA isolation Kit (Roche), and cDNA was synthesized with the Transcriptor First Strand cDNA Synthesis kit (Roche), both according to the instructions of the manufacturer. cDNA synthesis was performed with the CommonUni12G (GCCGGAGCTCTGCAGATATCAGCGAAAGCAGG) primer specific for influenza A vRNA. Next, all eight genomic segments were amplified in one reaction with Phusion High Fidelity polymerase (Thermo Scientific) using primers CommonUni12G and CommonUni13 (GCCGGAGCTCTGCAGATATCAGTAGAAACAAGG)^12^,^32^.

### Illumina MiSeq library preparation and sequencing

500 ng of the PR8-NS1(1-73)GFP virus RT-PCR product or the pHW-NS1(1-73)Dmd-GFP-NEP plasmid was sheared with an M220 focused-ultrasonicator (Covaris) set to obtain peak fragment lengths of 300–400 bp. Next, the NEBNext Ultra DNA Library Preparation kit (New England Biolabs) was used to repair the ends and to add the Illumina MiSeq-compatible barcode adapters to 100 ng of fragmented DNA. The resulting fragments were size-selected using Agencourt AMPure XP bead sizing (Beckman Coulter). Afterwards, indexes were added in a limited-cycle PCR (10 cycles), followed by purification on Agencourt AMpure XP beads. Fragments were analysed on a High Sensitivity DNA Chip on the Bioanalyzer (Agilent Technologies). The multiplex sample was heat denatured for 2 min at 96 °C before loading on the Illumina MiSeq chip. After the 2 × 250 bp Illumina MiSeq paired-end sequencing run, the data were base called and reads with the same barcode were collected and assigned to a sample on the instrument, which generated Illumina FASTQ files (Phred +64 encoding).

### Data analysis

The downstream data analyses were performed on the resulting Illumina FASTQ files (Phred +64 encoding) using CLC Genomics Workbench (Version 7.0.3) following the analysis pipeline as described in Van den Hoecke *et al*.^12^. The trimmed and filtered reads were aligned to the PR8-NS1(1-73)GFP reference genome (based on the plasmids used to generate the recombinant PR8 virus, with addition of the extra 20 nucleotides present at the 5′ site in the RT-PCR primers) or the plasmid reference sequence using the following parameters: match = +1; mismatch = −2; insertion/deletion = −3; length fraction = 0.9; similarity fraction = 0.8; non-specific match handling = ignore^12^. For the ‘Large Gap Mapper’, the same mapping parameters were used, together with the default ‘Large Gap Mapper’ settings, allowing large gaps in the mapping.

## Additional Information

**Accession codes:** The raw sequencing data can be found in the NCBI Sequence Read Archive with the accession numbers SRP052023 (virus sample) and SRP062322 (plasmid samples).

**How to cite this article**: Van den Hoecke, S. *et al*. Illumina MiSeq sequencing disfavours a sequence motif in the GFP reporter gene. *Sci. Rep.* **6**, 26314; doi: 10.1038/srep26314 (2016).

## Supplementary Material

Supplementary Information
