# Supplementary material for: Illumina MiSeq sequencing disfavours a sequence motif in the GFP reporter gene
Source: Sci Rep. 2016 May 19;6:26314. doi: 10.1038/srep26314 (PMC4872057; doi:10.1038/srep26314)
Supplement: Supplementary Information [file srep26314-s1.doc]

**Supplementary Information**

**Illumina MiSeq sequencing disfavours a sequence motif
in the GFP reporter gene**

**Silvie Van den Hoecke^1,2^, Judith Verhelst^1,2^ and Xavier Saelens^1,2,*^**

^1^ Medical Biotechnology Center, VIB, Ghent, B-9052, Belgium

^2^ Department of Biomedical Molecular Biology, Ghent University, Ghent, B-9052, Belgium

* Correspondence: xavier.saelens@vib-ugent.be


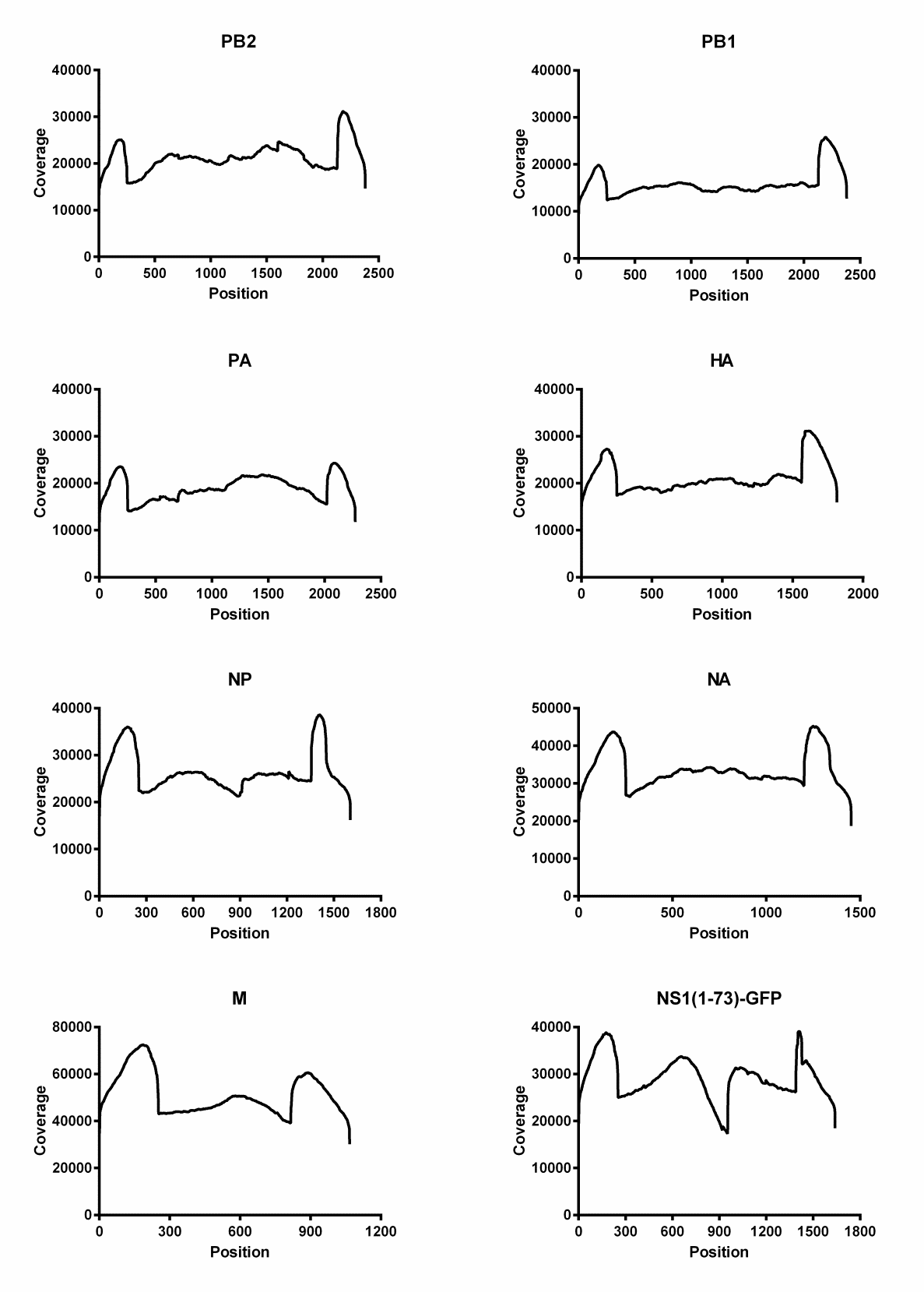
**Supplementary Figure S1**. Sequence coverage of the PR8-NS1(1-73)GFP virus. Sequence coverage for the different genome segments of the virus stock determined by Illumina MiSeq sequencing after Covaris shearing and CLC Genomics Workbench version 7.0.3 data processing. The obtained sequences were filtered, trimmed and mapped to the reference genome based on the eight plasmids used to generate the recombinant PR8 virus (with addition of the extra 20 nucleotides present at the 5′ site in the RT-PCR primers)^12^.


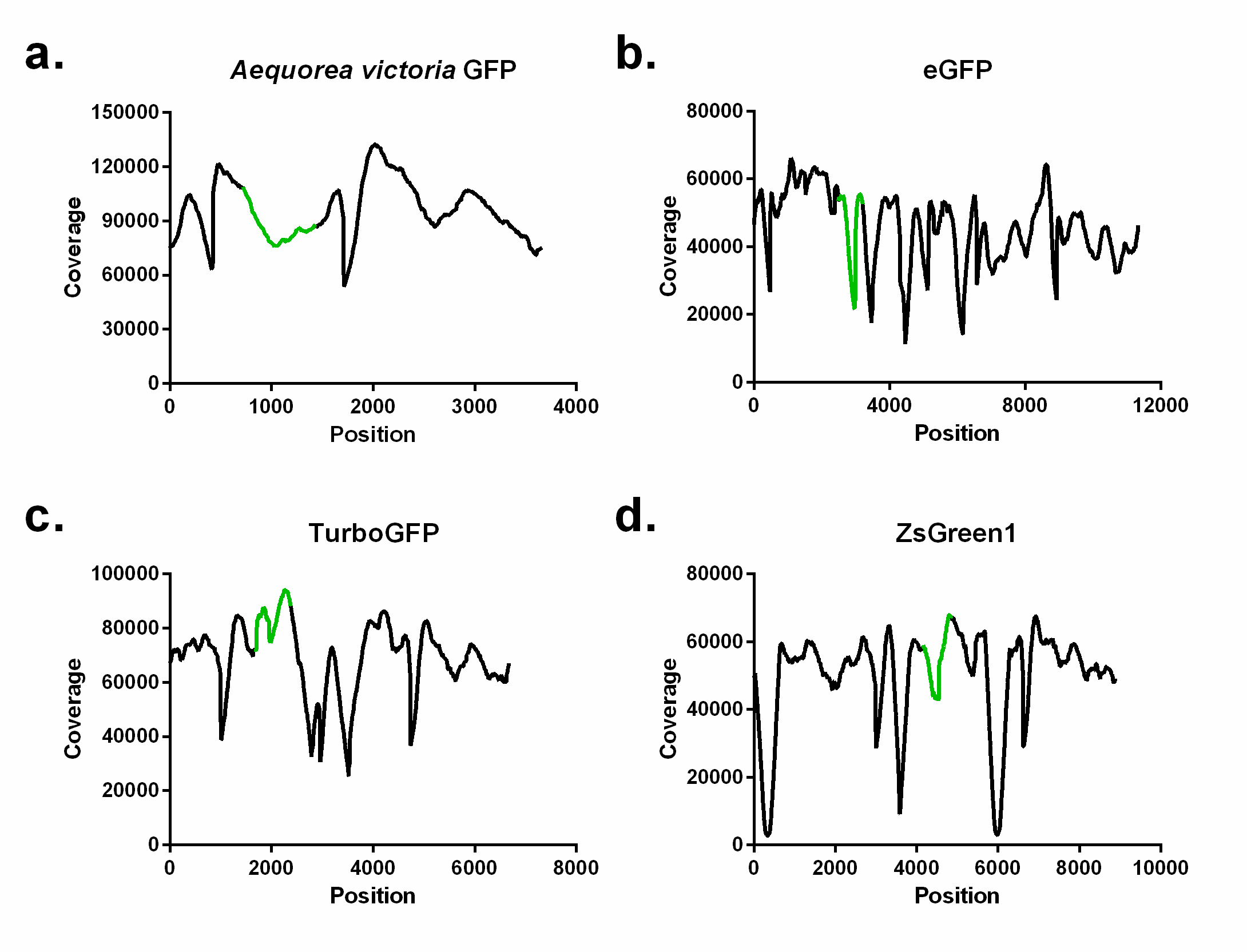


**Supplementary Figure S2**. Sequence coverage of plasmids expressing different GFP variants. The sequencing coverage is plotted in function of the nucleotide position in the plasmid containing the coding sequence for *Aequorea victoria* GFP (pBluAGFP^24^) (a), eGFP (pDG2-hRIPK4-WT-EGFP-puro^23^) (b), TurboGFP (pEF6-turboGFP-MCS) (c) and ZsGreen1 (pLVX-EF1a-IRES-ZsGreen1) (d). The position of the sequence coding for GFP in the different expression plasmids is marked in green on the coverage plot. Samples were sequenced on Illumina MiSeq (2*250bp) after Covaris shearing, followed by CLC Genomics Workbench version 7.0.3 data processing and mapping of the reads to the plasmid reference sequence^12^.
